# Registered nurses experiences of managing depressive symptoms at care centres for older people: a qualitative descriptive study

**DOI:** 10.1186/s12912-019-0368-5

**Published:** 2019-09-05

**Authors:** Gunilla Borglin, Kristina Räthel, Helena Paulsson, Katarina Sjögren Forss

**Affiliations:** 1 Department of Care Science, Faculty of Health and Society, Malmö University, SE-205 06 Malmö, Sweden; 2 Department of Nursing Education, Lovisenberg Diaconal University College, 0456 Oslo, Norway; 3 Department of Geriatric, Skåne University Hospital, SE-221 85 Lund, Sweden; 4 Åstorp Primary Care Centre, Region Skåne, SE-265 34 Åstorp, Sweden

**Keywords:** Content analysis, Care centres for older people, Nursing, Qualitative research, Registered nurses

## Abstract

**Background:**

Depressive symptoms and/or depression are commonly experienced by older people. Both are underdiagnosed, undertreated and regularly overlooked by healthcare professionals. Healthcare facilities for people aged ≥75 years have been in place in Sweden since 2015. The aim of these care centres, which are managed by registered nurses (RNs), is to offer care adjusted to cater to the complex needs and health problems of older people. Although the mental health of older people is prioritised in these centres, research into the experience of RNs of depressive symptoms and/or depression in older people in this setting is limited. Therefore, this study aimed to illuminate RNs, working at care centres for older people, experience of identifying and intervening in cases of depressive symptoms.

**Methods:**

The data for this qualitative descriptive study were collected through interviews (*n* = 10) with RNs working at 10 care centres for older people in southern Sweden. The transcribed texts were analysed using inductive content analysis.

**Results:**

The participants’ experiences could be understood from four predominant themes: (1) challenging to identify, (2) described interventions, (3) prerequisites for identification, and (4) contextual influences. Key findings were that it was difficult to identify depression as it often manifested as physical symptoms; evidence-based nursing interventions were generally not the first-line treatment used; trust, continuity and the ability of RNs to think laterally; and the context influenced the ability of RNs to manage older people’s depressive symptoms and/or depression.

**Conclusions:**

The process of identifying depressive symptoms and performing an appropriate intervention was found to be complex, especially as older people were reluctant to present at the centres and provided obscure reasons for doing so. A nurse-patient relationship that was built on trust and was characterised by continuity of care was identified as a necessary prerequisite. Appropriate nursing interventions—afforded the same status as pharmacological treatment—are warranted as the first-line treatment of depression. Further research is also needed into efficacious nursing interventions targeting depressive symptoms and/or depression.

## Introduction

Depressive symptoms and depression are among the most common mental disorders in older people [1]. Yet, both are underdiagnosed, undertreated and regularly overlooked by healthcare professionals or are simply regarded as inherent to ageing [2]. However, it is not a natural consequence of ageing [2], and the ability to treat depressive symptoms and/or depression successfully is high, even in older people. Despite this, older people have been demonstrated to be less likely to receive an intervention for depression compared to the rest of the population [3, 4]. They tend not to complain about feeling depressed [5] as this is perceived to be a sign of weakness [6]. In addition, depression is regarded as a mental illness and is stigmatised. Accordingly, older people avoid seeking help for depression, preferring instead to obtain help for physical disorders [6]. Depressive symptoms and depression include a number of signs and symptoms; for example, anxiety, various somatic symptoms and changes in function [7]. The identification can therefore be challenging for healthcare professionals; yet, it is known that depressive symptoms have a significant negative impact on the quality of life and sense of wellness of older people [8–10]. Depression is also associated with an increased risk of morbidity, suicide, decreased cognitive and social functioning [8–10].

Registered nurses (RN) are on the frontline of care. Therefore, they are well placed to identify, assess and intervene (i.e. in accordance with the nursing process) against depressive symptoms among older people [11, 12]. RNs have considerable potential to reduce the effects of depressive symptoms through the use of relevant, timeous evidence-based interventions. However, they seldom identify depression in older people [4, 13]. A lack of knowledge of depressive symptoms and an absence of confidence in RNs is a plausible explanation as to why RNs don’t readily discuss depression with patients [13, 14]. Another explanation is that RNs do not believe that this area falls within the scope of their practice [4]. Regardless of the setting, RNs were shown to have significant difficulty in correctly identifying depression in older people, but they were nevertheless at least as accurate as physicians in doing so, according to a recent meta-analysis performed by Mitchell and Kakkadasam [15]. The importance of identifying depressive symptoms and/or depression in older people and implementing appropriate interventions, as well as providing evidence-based care at all times, cannot be underestimated. An increasing number of older people are living for a longer duration while also continue residing in their own homes. Consequently, RNs in the primary care setting have a particularly important role to play in the delivery of services to older people with depressive symptoms and/or depression.

Care centres for older people (CCOP) have been in place in the Swedish primary care context since 2015 and were initially proposed by Akner, who suggested that the establishment of such centres nationally would address the gap in catering to the complex healthcare challenges of older people [16]. The services and facilities provided at these centres are complementary to those on offer at traditional primary care institutions. Certain criteria have to be meet for a primary care centre to become an accredited CCOP. They have to (1) offer a staffed telephone without push-button dialling and be reachable for a minimum of 15 h per week, (2) offer regular follow-up home visits to people aged ≥75 years in greatest need of the service, (3) offer coordinated individual care plans, (4) actively request the perspectives of patients and their significant others on all aspects of care planning, (5) offer preventative and health-oriented services and (6) employ at least one district RN or an RN with adequate specialisation in the care of older people.

The centres differ from the usual primary care institutions in that they are a RN-led service with a focus on medication reviews and nutritional and mental health. They also vary by offering longer appointments (i.e., 45–60 min) in contrast to the standard 5–15 min on offer in regular primary care. The centres are still under development particularly in regards to their organisation of services as well as to the RNs role, tasks and function. The principal aims of these centres are the provision of rapid access to health care and the delivery of optimal care adjusted to the complex needs of older people. The mental health of older people is considered to be a priority. Nonetheless, research into the experiences of RNs and their ability to identify depressive symptoms and/or depression in older people and implement appropriate treatment is limited in this setting. To our knowledge, this is one of the first study exploring this. Thus, this study aimed to illuminate RNs, working at CCOPs, experience of identifying and intervening in cases of depressive symptoms among older people.

### Design and methodology

A qualitative, descriptive design was adopted. The data were collected through interviews [17] and an inductive content analysis was performed, as described by Elo and Kyngäs [18].

### Study setting

The study was conducted in spring in 2018. The study participants were RNs employed at 10 different care centres for older people in southern Sweden. This model of care for older people was initiated in this region. Approximately 100 CCOPs existed in this region in 2018. This type of health service is not mandatory. Accordingly, the number of centres varies depending on the region, and many of them are still being developed.

### Sample and recruitment

Convenience sampling was applied [17]. Ten RNs agreed to participate. Eligible RNs had to have at least 1 year’s experience working for and holding a position at a care centre for older people. A study information letter was sent out via electronic mail to the operation managers at 37 centres. Initially, five centres agreed to take part, while a further nine cited a lack of time as the reason for not participating. A reminder was sent to the remaining 23 centres. A further three agreed to participate. Thereafter, no more replies were received. Two of the non-responsive centres were contacted once more and agreed to participate after receiving additional information (*n* = 10). All of the participants were women and had worked for 1–4 years at the relevant centre. One of the participants was a general RN, while the remainder held specialist qualifications (district RNs, *n* = 6; RNs who specialised in the provision of care to older people, *n* = 3).

### Data collection

The data were collected through interviews [17]. The interviews were based on one key question: ‘Can you please tell me about a situation in which you (as an RN at a CCOP) have been in contact with older people with depressive symptoms?’ Probing i.e. ‘Can you please give me an example?’ or ‘Can you please tell me more?’ was used, when necessary. The key question was initially tested on two RNs at two different CCOPs, to ensure its relevance and understandability in relation to the study objective. As this did not lead to any changes, the data from the test interviews were included in the analysis. The interviews, which took place at the workplace of the RNs, were digitally recorded and transcribed. On average, they lasted between 30 and 50 min and were conducted by either the second or third author. After eight interviews no new information was provided, but a further two interviews were conducted to ensure redundancy [19].

### Data analysis

The transcribed texts were analysed using inductive content analysis [18]. Inductive content analysis is said to be particularly useful when only a few studies deal with the phenomenon in *foci* or when the available research is perceived to be fragmented [20]. Content analysis per se relates to the study context, consequences, intention and meaning [21]. It is perceived to have several benefits, including the provision of a content-perspective, and also offers flexibility regarding the research design [22, 23]. First, the transcribed material was read several times in order to ensure that the researcher was immersed in the data [18]. Second, key issues (i.e., meaning units) were condensed and coded manually using colouring pens. Third, the different coloured codes were then interpreted and compared for similarities and differences. Fourth, they were sorted into tentative subthemes, without losing their content [18]. In the initial part of the analysis the second and third authors took the lead. However, all the authors separately read, analysed and discussed the text during the whole process to enhance the best possible account of the meaning found in the texts. Fifth and final, the authors agreed on 11 subthemes and four themes that unified the content within the subthemes were formulated. These were (1) challenging to identify, (2) described intervention, (3) prerequisites for identification, and (4) contextual influences. These themes illuminated variations in the experience of RNs in identifying and intervening in cases of depression among older people (Table 1). The qualitative methodology applied and subsequent reporting of the results adhered to the consolidated criteria for reporting qualitative research [24].

**Table 1:** Example process of analysis

Meaning units	Condensed code	Code	*Sub-theme*	Theme
Many older persons are ashamed of feeling blue, it is taboo and they don’t want to talk about it. This comes from how it was before; it was different compared to today and some still feel ashamed not feeling ok or going to the counsellor, it is very embarrassing. (RN3)	Older persons ashamed, taboo topic, and won’t tell. Feeling ashamed and not being ok with counselling.	Difficulties confessing the experience low mood.	Not seeking help	Challenging to identify
I guide them … I send them on to home care, the physiotherapist, the dietician and so on I try to see the whole person. (RN1)	Guiding them, offering the help they might need and aiming to see the whole person	Guidance and support.	Offering support	Described interventions
But I can put forward the question ‘do you feel blue’ and I can ask the question, ‘why are you prescribed sleeping tablets I am not afraid of that as there is nothing strange about that. (RN10)	Nothing strange about asking a direct question about low mood and medication	Natural as an RN to ask difficult questions	Having the courage to ask	Prerequisites for identification
I am grateful that I can book longer appointments for my patients as not only can it pick up signals about depressed symptoms but about other stuff too. (RN10)	Grateful for being able to book long appointments which means being able to pick up depressed mood	Time of essence in picking up low mood	Time	Contextual influences

## Results

Four themes were identified and considered to reflect the participants’ experience. The identified sub-themes are in italic for clarity.

### Challenging to identify

It was challenging identifying depressive symptoms and/or depression in older people. Particularly since many of the older people *did not seek help* as they considered depression to be normal consequence of ageing. The participants suggested that depression was not always identified in older people:

I believe it is an underdiagnosed group … They don’t come and ask for help. Instead, they believe [that] this is how it is; it’s the trajectory of life, but they can actually get help. (RN3)

I think there are a lot of older people who we miss; that is what I think … . How to do it? I don’t know [identifying them]. (RN5)

The participants were also of the opinion that older people were ashamed that they were depressed:

When they grew up, mental health problems were not spoken about and, if they existed, they were hidden away. I think then it is much harder to take the step [to ask for help] … They never hit the wall in those days; no, they found the door … . (RN2)

In general, the perception was that overall, older people seemed to cope better with impediments than younger ones. It was not uncommon for relatives to contact the centre to expresses their concern:

The younger generation calls for help [an appointment] for the slightest adversity, needing counselling because their cat died … I don’t think the older generation do that. They seem to handle adversity; they are more used [to it], and they just seem to suck it up. It is not until their significant other reacts [to say] that it is not working at home. But no, it is not common that they call for help [for depressive symptoms]. Instead, they call for help about something else … . (RN9)

Participants at the centres described no routine of the use of validated instruments. They reported that the only time standardised assessments were used (i.e., the Geriatric Depression Scale [GDS]) was when they performed a memory assessment or on the recommendation of a primary care physician [PCP]:

GDS is only used for a memory assessment, but that is a thought. I can do that; nothing says I can’t do that [use it on other occasions too]. (RN10)

The participants explained that older people often gave another reason (other than depressive symptoms) for their visit to the centre:

… They arrive and have something else they wish to bring up. A bit like, yes, a cover for what they really want to bring up. (RN4)

The study subjects found it difficult to identify the cause of depressive symptoms, for example, to distinguish between physical, social or psychological symptoms. The most prominent symptoms were insomnia, a diminished appetite and pain:

It is very common that they seek help for a physical symptom … but it might not be the pain in their leg that is their biggest issue but rather the problem with not being able to sleep. (RN6)

Mainly, they contact us for a physical complaint … I do not believe that we are very good at identifying them (depressive symptoms). I don’t think so, if I’m bluntly honest with you. (RN2)

Some older people repeatedly sought treatment, either for the same physical health complaint or others. This behaviour alerted the RNs to search for other causes:

There are those repeat callers. Then, I book an appointment, for example, for their blood pressure, and I book a longer time than what I do for younger patients. Then, I do the routine [check-up] for blood pressure, and after that, I take time to [ask] if there is actually something else … ‘Do you feel sad?’ … ‘Do you have trouble sleeping?’ (RN10)

The ability to map the individual’s situation and amalgamate different pieces of information to form a coherent whole was considered to be very challenging (i.e., akin to completing a jigsaw puzzle), but the participants thought that it was necessary to gain a holistic impression and establish whether the complaint was depressive or physical in nature:

It is about the conversation and meeting the older person, so one is not only focusing on what they are seeking help for … one sees more the entirety of the situation. It concerns not just, for example, what sanitary pads the patient should have, but rather the full picture … what the problem is. (RN9)

The ability of the study subjects to identify depressive symptoms was thought to be influenced by the context in which they met the patients. Home visits were viewed to be the most beneficial as it was easier to identify depressive symptoms in a home than in a clinical setting:

It can be the blinds [are] down, in darkness almost; they don’t want to leave the house... and yeah, with little more than old food. One often sees that they have received a portion of food, but they only finish half of it, so they can eat the rest in the evening; this is not uncommon. (RN7)

Face-to-face meetings were considered more valuable than telephone conversations in identifying depressive symptoms as body language, facial expressions and postures signified that something was wrong:

Meeting up with the patient is [our preference]. You have facial expressions, how they present themselves and, ehm, clothes, hygiene and smells … One cannot identify anything on the phone … if they themselves [say] that they are feeling blue, okay; but when they [refer to] something else, then one cannot identify it that easily. (RN9)

### Described interventions

*Support* and *follow-ups* of pharmacological treatments were the first-line approach to the treatment of depressive symptoms in older people, with antidepressants constituting the most common intervention. The participants felt obliged to refer older people to a PCP:

I book an appointment with their PCP … who prescribes mirtazapine [an antidepressant], and after some weeks, they are back and feeling like a new person. (RN10)

Although pharmacological treatments were considered to be an efficacious first-line intervention, nursing interventions were also considered to be as valuable. A combination of antidepressants and a nursing intervention was described as vital in supporting older people with depression:

… and here, we have a really good role to fill because often you are focused on their needs—their medication and this and that—but if they don’t eat and they sleep all day, then a nursing intervention needs to come first. (RN8)

Support, in the form of advice with regard to dietary intake, sleeping and physical activities, was perceived to be as vital as an antidepressant agent in treating depressive symptoms:

I think this is also a part of [the] motivation, because one often hopes and trusts that a pill will solve everything. But it doesn’t; not without sleeping, eating and socialisation. There are several building blocks, and they are all equally important. One can take as many mirtazapine [an antidepressant] as one likes, but if one continues to remain in solitude and with the blinds down, one will not become well. (RN8)

Offering general support (i.e., listening, taking time to communicate and counselling) was perceived to be an appropriate intervention:

When it concerns older persons, I think that they may take some time before they actually decide to come … then I have to invest some time and most importantly listen … I want to be able to have time for talks, and then it is very important to take time, to listen and confirm …. (RN4)

It was suggested that additional time should be found in care situations with older patients.

I think they might feel unwell as they are alone and don’t have anyone to talk to. So, for example, when I dress their feet, then I usually also lubricate and massage their feet, as I have learnt tactile massage, so I take some additional circuits and massage every little toe … I don’t have time, but make the time. (RN10)

Lack of competence in dealing with certain types of support (i.e., counselling) was an impediment to the initiation of a psychological intervention for certain people. By contrast, others thought that counselling could be performed by anyone:

Time is available, so I don’t feel limited in that sense, but it is maybe my knowledge. Maybe, I need more knowledge about depression and older people. (RN9)

There are studies about counselling, and it does not actually need to be done by a trained counsellor as it is more about confirmation and allowing the person to talk through the situation. (RN3)

Feelings of inadequacy were associated with a belief that the only available strategies with which to manage depressive symptoms were general support and counselling:

I would like that we had plans, some type of guideline on how to manage it. Is there something to do about it before one prescribes antidepressants? I don’t think these older people should need to have so much medicine. (RN6)

Preventing the interpretation of possible failure as a personal defeat and supporting the self-image of the older patient’s self-image was considered to be vital:

One has to take one step at a time. They cannot begin to socialise, exercise and eat; not all at once. Instead, the most important thing is that they eat, and when [their] energy returns, the next step can be taken so that they don’t get performance anxiety. That would be terrible as they would experience failure every time. (RN8)

Other healthcare professionals; for example, dieticians, physiotherapists and pharmacists, were perceived to be of considerable importance to the team. This meant that the participants could suggest physiotherapy, for example, as a means of catering to a social rather than a physical need:

It can also be that one looks beyond the loneliness and sees a physical need. One can offer physiotherapy as we provide group physiotherapy here. Then, one can broach the topic stealthily, instead of [saying]: ‘Here is an intervention, as you are lonely’ … as it is a bit shameful to be alone. (RN8)

Follow-up consultations via home visits were used if there was uncertainty as to whether or not older people were coping with daily life. Home visits provided them with a more complete picture of the situation and helped them to identify the type of support needed:

... and in their home, you see, does it work? Are they dressed and washed? Does it look like they are coping with their personal hygiene when I arrive [at their home], or have they not bothered? (RN8)

Drug treatment follow-ups were perceived to be part of the supportive role provided by RNs, while others thought that this was the responsibility of the PCP alone:

Actually, I do it to support the PCP who is the one who is supposed to follow-up drug treatments … one keeps contact with the patient and checks how the medication is working or if it not is working. (RN3)

Unfortunately, my role at present does not work like that. It is enough that I think that someone suffers from depression to forward it to a PCP so that the patient gets an appointment. But I do not follow it up afterwards. (RN2)

### Prerequisites for identification

Prerequisites to the identification of depressive symptoms and/or depression and the subsequent implementation of interventions included the importance of creating a *trusting relationship*, having *the courage to ask*, possessing *knowledge* and offering *continuity*.

A central prerequisite was the establishment of a relationship built on trust and for the RNs to be perceptive and understanding. The importance of trust was emphasised:

Most important is to gain their trust, otherwise it doesn’t matter what I say. I usually start by listening and confirming … they are allowed to talk, and I acknowledge that they are feeling like that [confirmation] … and when they feel that they are being heard and seen, then I try to explain, and carefully add what I think and how we might proceed. You do not need to throw in words like ‘depression’. You can talk around it. Talk about solutions instead of problems ... . (RN4)

Relevant information could be identified, both at the beginning and at the end of conversations and, from what was not mentioned: ‘The silence of thinking is very good; then you get a lot of answers’ (RN10). In addition to forming a trusting relationship, having the courage to ask older people about their mental health was considered to be vital when identifying depressive symptoms:

I ask the question directly. But it is not the first question. Rather, we talk for a while, so then it is not that hard to ask the question … You talk about [their] mood instead of asking: ‘Do you feel depressed?’ That is a slight nuance. (RN1)

Directly asking older people ‘Are you depressed?’ rarely occurred. Insufficient knowledge about how to deal with the response was given as the reason; suggesting implied reluctance to identify depressive symptoms:

I think that I have never posed the question [in a] straightforward [way]: ‘Are you depressed?’ No, I haven’t. It is maybe because I don’t really have a good strategy for moving forward ... If I look in the booking system, I have no time for appointments … it might have the effect that one doesn’t want to identify anything since I have nothing to offer. Ouch, it sounds horrible … . (RN2)

Knowledge and experience were thought to be fundamental to managing depressive symptoms:

Maybe, I need more knowledge about older people and depression. However, I feel it is enough to identify them [symptoms] … but that might be wrong; maybe I have missed a lot [the symptoms of depression]. (RN9)

It was proposed that older people with depressive symptoms needed a care manager who could provide continuity in nursing care specifically as, in the experience of the participants, important care was otherwise missed:

I cannot take care of them all, so then they end up at the RNs at the primary care clinic, where surely, they get the same care. I am not criticising this, but they do not get continuity. There are several different RNs there, and they take turns doing different jobs. So, I can say that one might miss some things as one does not get continuity, which is a pity, because I think this is important. (RN6)

### Contextual influences

Organisational *structure*, *time*, *accessibility* and *collaboration* were seen to influence the participants experience of identifying depressive symptoms. Working at the interface between the primary care centre and the care centre for older people, coupled with a lack of designated people to answer the telephone, was described as challenging:

They [depressive symptoms] are a bit more difficult to catch up with as we have different RNs answering the phone. One could, of course, read the material in the case notes if they have called a lot, but one doesn’t always track back. So, it is not collected as it would have been if it were the same person answering. (RN2)

Substantial frustration with organisational changes was voiced by the study subjects, who felt that the changes had a detrimental influence on the centres. Their working schedule, effective from 1 January 2018, primarily consisted of the coordination of care plans in relation to admissions from secondary care:

I don’t know if you heard … the new law about coordination, yeah, that has become a huge thing for us, and I am the one sitting here and coordinating this at the centre; and at the moment, all the focus and time at the care centre for older people is, unfortunately, taken [up] by this. (RN3)

As a result of their new obligations, the participants perceived themselves to be performing tasks on a daily basis that did not utilise their competencies:

We would like the care centre for older people to be completely different to what it is today. Today, we are mainly engaged in care plans, which is highly time consuming, and we receive a huge amount of faxes from the home care RNs that we are supposed to administer. That needs to be changed. We can’t have it like this. We need to take care of our patients; not to act as the postman. (RN9)

Time needed to be calculated into the equation to successfully identify and treat depressive symptoms. The participants believed that it was critical that they were in charge of accessibility and appointments:

So, there we are, we have an important role, I believe … we have contact [with the older person, which] the PCPs don’t have time for. (RN5)

The general lack of relevant guidelines and/or pathways within organisations was perceived to be a limitation, and the participants had a number of questions that they wanted answered:

What can I do before possible drug treatment? How long should an older person be treated with drugs, and when should the counsellor be established? (RN6)

Ease of access to professionals (counsellors or psychologists) differed between the centres. The general experience was that the provision of such therapy was not prioritised for older people:

But when you compare older people with younger ones [regarding the provision of therapy], then pharmacological treatment is, above all else, for older persons. (RN3)

The participants were frustrated about the lack of habitual access by older patients to reasonable resources. Some of them were mandated to refer the patient to a psychologist, while others had to go through a PCP to obtain a referral:

Though I cannot solve everything, I can forward it. I become a bit like a spider in a web and can send them on [to relevant support] (RN1).

I am not allowed to refer the patient to a psychologist, counsellor or the memory clinic. I cannot call and consult anyone; or maybe I can, but I do not have a real mandate to do so … at times, this feels like a waste of resources—needing to go through the PCP—but that is how the healthcare system works. (RN6)

Collaboration between professions and organisations was regarded as fundamental to supporting this group of patients. Positive and negative experiences were narrated in this regard:

We are not a group of professionals who meet the patient simultaneously, but we do different parts of the whole, and we have different competences and professions, but we do work towards the same goal anyway. (RN1)

I am working on developing much better collaboration with home care; it is rather tricky, but we are working at it ... and on others, such as a social care caseworker as we [do] not have a direct line and no contact space, and where it would be much easier if I could just contact them … but we have not been successful in establishing it. (RN6)

There was consensus that being able to offer relevant services to older people demanded teamwork. The PCPs were regarded as playing a central role in highlighting the importance of nursing care and nursing interventions and in demonstrating that these interventions were of as valuable as drug treatments:

What I would like to highlight more is the role of RNs, because we are also all very medically focused. And we should be more in the team. I think that [it] would have been much better—when patients see the doctor for their medication—for the doctor to show this [the nursing intervention] [to be] equally important [i.e., to the medication]. I also care just as much about your eating, sleeping and socialising; we are equally important components. (RN8)

## Discussion

This study aimed to illuminate RNs, working at CCOP, experience of identifying and intervening in cases of depressive symptoms among older people. Their experience could be understood from four themes; (1) challenging to identify, (2) described intervention, (3) prerequisites for identification, and (4) contextual influences, illuminating the variations in the experience.

It was noteworthy that a systematic assessment of depressive symptoms was not part of routine nursing care at the centres. Particularly considering that the older people were less likely to seek care for depressive symptom and more likely to use covert reasons for doing so. Ahuriri-Driscoll et al. found that the use of standardised screening tools and evidence-based treatment guidelines had a significant impact in moderating depression in older people [25]. The relative importance of conducting systematic physical, psychological and social assessments when assessing older people for depression has also been proposed [26]. Interestingly, in the current study, the GDS was routinely used by the RNs for memory assessments, but its usability for other purposes was not an entrenched aspect of care. The use of GDS has been demonstrated to be invaluable in identifying depression and depressive symptoms in older people [27–29]. Smalbrugge et al. described its usefulness in making relevant nursing diagnoses and identifying appropriate interventions for use in treating depressive symptoms, as well as its suitability when following-up and evaluating the effects of the actions taken (i.e., nursing interventions or pharmacological treatment) [28]. A reasonable action to remedy the lack of standardised screening could be to implement a nursing process according to which subjective (i.e., the patient’s experience) and objective data (i.e., validated instruments and assessments) are collated to ensure quality care for this population. The nursing process could constitute a framework that would be used to address individual patient needs [30]. Hence, it is likely that the use of a relevant problem-solving and decision-making model such as the nursing process would assist RNs to identify depressive symptoms and/or depression. It is imperative that a systematic structure is implemented to assist with the identification, particularly as older people tend to seek care for a wide array of physical symptoms rather than for feeling depressed, as evidenced in the current study and elsewhere [31–33]. The establishment of a systematic process would also facilitate the ability of RNs to amalgamate different pieces of information in order to visualise the bigger picture. Chew-Graham et al. [34] corroborated the value of being able to perceive a situation holistically, that is, to understand the underlying factors that influence the mental health of older people and to be able to offer relevant interventions accordingly.

Appropriate nursing interventions for depression in older people were not the first-line treatment approach taken by RNs at the care centres. There was consensus among RNs that the first-line approach was to book an appointment with a PCP, who, in the majority of cases, would initiate pharmacological treatment (i.e. selective serotonin reuptake inhibitor - SSRI). The evidence suggests that the majority of older people do not respond well to treatment with antidepressants. In other research, the effect of SSRIs has not been demonstrated to be superior to that of placebo [35–37]. It is instead recommended that nursing care and interventions should, if medical contraindications do not exist, constitute the first-line treatment for depression in older people [38]. Nursing interventions that target the fundamentals of care, such as nutrition, rest, sleep and social activities, [39] were demonstrated to be as important as pharmacological medications in treating depressive symptoms in the current study. Thus, in contradiction to the approach of referring older people to a PCP to receive pharmacological treatment. However, the current evidence base is still limited regarding psychosocial interventions that target depressive symptoms in older people [40]. That said, some evidence exists concerning the importance of engagement in social activities [40]. This highlights the importance of RNs adopting strategies that include listening and talking to older people and supporting them to engage in social activities, as a part of beating their blues. Silva et al. state that RNs have an important role to play by listening to and engaging older people to be involved in their treatment, as well as strengthening their self-esteem [29]. Perhaps more importantly, enhancing their sense of belonging despite old age [41]. Our findings, as well as others, indicate that greater knowledge is warranted about effective interventions that target depressive symptoms [40, 42]. In particular, nursing interventions developed to reflect daily clinical practices and that consider the complexity of the human condition [43]. Such knowledge would support the ability of RNs to develop and implement evidence-based practices to treat depressive symptoms and/or depression at the centres.

Our findings reflect the value of RNs spending adequate time with older people and offering them continuity of care, as well as the establishment of a professional nurse-patient relationship that is based on trust. The latter was evidently a prerequisite to the identification of depressive symptoms in older people by RNs and the implementation of subsequent interventions in the current study. The importance of trust is emphasised by Belcher and Jones [44], who identified trust as central to the creation of a sound nurse-patient relationship. A good-quality relationship between an RN and a patient is undoubtedly essential to nursing [45–47]. It is regarded as an important factor for the participation of patients in their care, as well as the ability of RNs to offer and deliver quality care [44, 48]. Besides the importance of establishing trust, the need for continuity of care and a care manager for older people was also highlighted by the current study findings. Following a systematic literature review, Gilbody et al. found that programmes for depression in which the role of RNs as care managers was enhanced and that featured regular follow-ups were beneficial for the management of a depressive mood and/or depression [49]. It is somewhat paradoxical that, on the one hand, trust and continuity were highlighted as key aspects of managing this population but, on the other hand, the findings also implied that asking them whether they felt depressed was not standard practice. Limited knowledge about how to deal with the possible answer and restricted ability to offer appointments were given as reasons for not asking direct questions. These findings are substantiated by Ell et al., who reported that home care RNs expressed reservations about asking patients direct questions pertaining to depression for fear that doing so would invade patient privacy or trigger patient distress. They also voiced concerns about contributing to the administrative burden [50]. This is not unique to RNs, Burroughs et al. reported that physicians were hesitant to ask older people whether they were depressed during time-constrained surgery appointments to avoid opening ‘a can of worms’ [51].

Care organisations are said to have less control over workflow than other services [52]. As a result, they experience a high degree of unpredictability [53]. This was obvious by the way in which RNs were obliged to administer coordination care plans, thus removing them from direct patient care at the centres. This meant that they could not make use of their competencies and detached them from their area of expertise—the mental health of older people. Thus, it is not likely that these organisations would be able to reduce and manage depression in older people based on the case findings of RNs. Organisational processes and structures are known to affect the working situation of RNs and the quality of care that is provided [54]. The inability to manage care threatens the quality and safety of care provision [55]. The need for formal management approaches at centres— that govern checklists, protocol, standards and pathways—was also raised by the RNs as these were considered to be effective tools for the coordination of care. In particular, when the quality and safety of health care ‘depends on ensuring that all the necessary elements to meet patient needs are aligned in the right place and at the right time’ (p. 4), the use of care pathways and protocols is pivotal to addressing the complexity of care-related processes and diminishing risks [53]. The complexity of identifying depression and implementing recommendations, as well as in the need for more guidance and support to address the needs of this patient group, has been corroborated elsewhere [56]. Additionally, it is not uncommon for healthcare organisations to lack the supportive infrastructure that is needed to treat depression effectively [57].

The importance of possessing the collaborative competencies needed in healthcare services [58, 59], where the latter often is characterised by discontinuity of care, was demonstrated in the current study. Access to relevant team members (i.e., psychologists), and close internal and external collaboration, were required to offer relevant services in this regard. Working in a multidisciplinary team to ensure continuity of care, patient safety and quality of care was one of six core competencies (i.e., standards of clinical care) that were identified as essential by the RNs [60]. Teamwork is also known to improve patient planning, as well as enhance clinical efficiency and support in person-centred care [61].

The current study findings raise questions about social justice and equal rights as the RNs reported that older people did not have equitable access to counselling compared to younger patients. Talking therapies, such as cognitive behaviour therapy, are vital to the treatment of depression [62, 63]. Psychological treatment is as effective for older as it is for younger people [64]. An increased risk of suicide has been reported for older persons who are depressed compared to younger people [65, 66]. Thus, access to relevant treatment by older people should be equal to or even greater than that given to younger people.

## Methodological considerations

The current study findings should be considered in the context of its limitations. The study design (i.e., qualitative descriptive) meant that emphasis was placed on the interpretation of the participants’ experiences [67, 68]. The participants in this study were selected using convenience sampling. This technique is considered suitable when recruiting readily available participants from a specific healthcare setting (i.e., care centres for older people) [17]. Although this study made a valuable contribution to the literature on the topic, it was based on a relatively small sample (*n* = 10), which could have implications regarding its trustworthiness. Nonetheless, the sample size was considered to be appropriate as we received guidance on the notion of response saturation after eight interviews, after which no new information emerged [69]. Homogeneity of the sample, in terms of gender and time employed at the centres, might also have affected the transferability of the results. However, data saturation occurred at a very early stage in the study by Guest et al., with 34 of the 36 developed codes being formulated from the first six interviews. They concluded that in studies with a high level of homogeneity among the participants, ‘a sample of six interviews may [be] sufficient to enable the development of meaningful themes and useful interpretations’ [68], (p. 78.). The data were analysed using inductive content analysis, which provided an opportunity to structure and present the results according to themes. There is always a risk of subjectivity in data interpretation as it is possible to interpret the text in different ways. To reduce this, the findings were discussed on an ongoing basis by the researchers during the analysis until high intersubjectivity agreement was reached. By describing the analytical procedure used and including the quotations, it is possible for readers to judge the conformability of the data as they inform how the findings were based on the participants’ opinions [17]. To the best of our knowledge, no studies regarding RNs experiences of identifying and intervening against depressive symptoms and/or depression have been published. Thus, this study can be considered to contribute with new knowledge to this field and particularly to nursing in contexts similar to the centre.

## Conclusion

The process of identifying depressive symptoms and/or depression in older people and recommending appropriate interventions was demonstrated to be challenging and complex in the current study. Particularly as the older people was reluctant to seek help for depressive symptoms and when they did, they provided obscure reasons for doing so. To effectively treat depressive symptoms, it is vital that a trusting relationship that is characterised by continuity of care is established between older people and RNs, while expertise in coordination and follow-up is also necessary. To be able to offer optimal and person-centered care, RNs need to have access to standardised guidelines and a clear organisational support structure. There is also a need for optimal interprofessional collaboration. More in-depth knowledge is required on effective and relevant nursing interventions for depressive symptoms and/or depression in order to see beyond the administration of pharmacological treatment, which is currently – and erroneously - the first-line treatment of depressive symptoms and/or depression in older people.

## Data Availability

The datasets generated and/or analysed during the current study are not publicly available due lack of consent of sharing raw material, but parts of the material can be available from the corresponding author upon reasonable request.

## Abbreviations

CCOP: Care Centre for Older People
GDS: Geriatric Depression Scale
HADS: Hospital Anxiety and Depression Scale
MADRS: Montegomery Åsberg Depression Rating Scale
NBHW: National Board of Health and Welfare
PCP: Primary Care Physician
RN: Registered nurses
SSRI: Selective serotonin reuptake inhibitor
